# Associations Between Genetic Variants in *MCT2* (rs3763980, rs995343, rs3763979) and *MCT4* (rs11323780) with Blood Lactate Kinetics Before and After Supramaximal Exercise

**DOI:** 10.3390/ijms26167865

**Published:** 2025-08-14

**Authors:** Ewelina Maculewicz, Aleksandra Bojarczuk, Andrzej Mastalerz, Monika Johne, Anna Mróz, Aleksandra Garbacz, Petr Stastny

**Affiliations:** 1 Faculty of Physical Education, Jozef Pilsudski University of Physical Education in Warsaw, 00-968 Warsaw, Poland; ewelina.maculewicz@awf.edu.pl (E.M.); andrzej.mastalerz@awf.edu.pl (A.M.); monika.johne@awf.edu.pl (M.J.); anna.mroz@awf.edu.pl (A.M.); 2 Department of Laboratory Diagnostics, Military Institute of Aviation Medicine, 01-755 Warsaw, Poland; 3 Faculty of Physical Education, Gdansk University of Physical Education and Sport, 80-336 Gdansk, Poland; 4 Faculty of Animal Genetics and Conservation, Warsaw University of Life Sciences, 02-787 Warsaw, Poland; aleksandra_garbacz1@sggw.edu.pl; 5 Faculty of Physical Education and Sport, Charles University, 162-52 Prague, Czech Republic; stastny@ftvs.cuni.cz

**Keywords:** genotype, genetic variants, lactate kinetics, athletic training, sprint, elite athletes, sub-elite athletes, physical activity

## Abstract

Despite progress in understanding the role of monocarboxylate transporters (MCTs) in lactate (LA) accumulation and removal, it remains unclear whether *MCT2* and *MCT4* variants enhance LA utilization. This study assessed associations between *MCT2* (rs3763980, rs995343, rs3763979) and *MCT4* (rs11323780) polymorphisms and LA concentration. A total of 337 male athletes from the Czech Republic and Poland, divided into elite, sub-elite, and physically active individuals, completed two all-out Wingate tests. Before these tests, DNA was collected and analyzed for single-nucleotide polymorphisms (SNPs). LA concentrations were measured before and after the tests. *MCT2* rs995343 showed the broadest associations. It was significantly associated with resting LA (LArest) in the overall cohort (codominant: false discovery rate (FDR)-adjusted *p* = 0.04; dominant: FDR-adjusted *p* = 0.03) and with peak LA concentration (LAmax), accumulation (ACC), and clearance (DCC) in the physically active group (all models: FDR-adjusted *p* = 0.02–0.04). *MCT2* rs3763980 was associated with LArest in the overall group (codominant and recessive: FDR-adjusted *p* = 0.04). *MCT2* rs3763979 was associated with LArest (FDR-adjusted *p* = 0.009–0.003) and LA30′ (FDR-adjusted *p* = 0.003–0.04) in the overall and physically active cohorts. *MCT4* rs11323780 was associated with LArest in elite athletes (recessive: FDR-adjusted *p* = 0.03) and with ACC in the physically active group (codominant and recessive: FDR-adjusted *p* = 0.03). These findings indicate that *MCT* polymorphisms contribute to variability in LA metabolism, influencing anaerobic performance and recovery.

## 1. Introduction

Physical activity in sports is a complex phenomenon heavily influenced by various interrelated factors, with genetics playing an important role [1]. Genetic predispositions are believed to contribute to individual differences in athletic potential and may influence overall performance outcomes [2]. Blood lactate (LA) plays an essential role in the metabolism during exercise [3]. Under anaerobic conditions, there is a marked increase in LA production and accumulation within the bloodstream, particularly at elevated levels of exercise intensity [4]. Several factors contribute to regulating LA production and clearance, with monocarboxylate transporter (MCT) genes, especially *MCT1*-*4*, playing a critical role in these processes. MCT1-4 exhibit a broad substrate specificity as they shuttle LA, pyruvate, and ketone bodies. MCT1 is ubiquitously expressed except in β cells of the endocrine pancreas [5], MCT2 in the liver, kidney, testis, and the brain, MCT3 within retinal pigment epithelium and the epithelial layer of the choroid plexus [6], and MCT4 in metabolically active tissues reliant on glycolysis and anaerobic pathways [7]. MCT1 has a lower affinity for LA (Km approximately 3–5 mM) than MCT2 (Km ≈ 0.7 mM). MCT1 and MCT2 are both responsible for the uptake of LA into cells, with MCT1 functioning more efficiently under elevated LA levels, while MCT2 enables effective transport even at low LA concentrations [6]. MCT3 has a Km of ~6 mM [8] and MCT4 20–35 mM [6]. MCT3 has a more specialized, epithelial function, and MCT4 is specialized for the efflux of large quantities of LA from cells exhibiting high glycolytic activity, such as fast-twitch skeletal muscle fibers. Slow-twitch muscle fibers (type I) primarily utilize oxidative phosphorylation for energy production [9]. Sprinting predominantly relies on fast-twitch muscle fibers (type II), which mainly depend on glycolysis [10]. During physical activity, plasma LA levels rise quickly, and it was traditionally thought that LA serves solely as a waste product, which is then transported to the liver, kidneys, or other tissues for removal [11]. However, it is now widely recognized that muscles metabolize LA [12]. Muscles play a significant role in the overall clearance of LA from the body during exercise [12,13]. Producing and accumulating LA is crucial for maximal effort, especially during approximately 30–90 s [14,15]. The assessment of blood LA concentration is one of the frequently measured parameters in clinical exercise evaluations and assessments of athletic performance [16,17,18]. Current exercise training methodologies rely on blood LA levels to assess physiological demands, providing insights into optimal exercise intensities for diverse populations [19]. It has been documented that short-term high-intensity exercise performance is correlated with post-exercise blood LA concentration [20]. It is also essential to highlight the influence of genes on LA concentrations in individuals who engage in high-intensity exercise. Recently, we showed that the TAC haplotype (rs3763980, rs995343, rs3763979) in the *MCT2* gene is associated with altered LA clearance in sprinters [21]. According to the SNP National Center for Biotechnology Information (NCBI) database [22], no publications are available regarding *MCT2* rs3763979. Rs955343 is described solely in the context of cancer patient prognosis [23,24], and rs3763980 relates to response to methotrexate [25,26]. There is also no available literature on the *MCT4* rs11323780 variant. The absence of literature linking *MCT2* polymorphisms (rs3763980, rs995343, rs3763979) and *MCT4* rs11323780 with LA utilization (accumulation and removal) dynamics presents a significant knowledge gap. The only relevant study currently available is by Maculewicz et al. (2025), which focuses on haplotypes and gene-gene interactions [21]. Therefore, this study aimed to assess the potential association between selected *MCT2* and *MCT4* polymorphisms with LA metabolism. Using SNP genotyping, we investigated the genetic predisposition for efficient LA utilization in elite, sub-elite sprinters, and physically active (control) men. We chose the sprinting discipline because LA production occurs when muscle activity surpasses its aerobic threshold [27].

## 2. Results

Sample sizes, ages, body masses, and heights across the elite, sub-elite, and physically active groups are summarized in Table 1.

The Hardy–Weinberg equilibrium (HWE) was confirmed for all genetic variants analyzed in this study, including *MCT2* (rs3763980, rs995343, rs3763979) and *MCT4* (rs11323780) (Table 2). No significant association was found in the analysis of a single locus.

The data for all LA concentration time points and performance-related parameters across genotypes and groups are presented in Supplementary Table S1. The following variables were included: LA concentration at rest (LArest), after warm-up (LAaftwarm), in the third minute of rest after the first but before the second Wingate test (LABETWEEN), immediately after the second Wingate test (LAaft2WAnt), and during recovery (LA3′, LA6′, LA9′, LA20′, and LA30′ minutes (min) after the second Wingate test). Additionally, maximum LA concentration (LAmax), LA accumulation capacity (ACC, the difference between LAmax and LArest), delta clearance capacity (DCC, the difference between LAmax and LA30′), and peak power output normalized to body mass (Pmax [W/kg]) were reported.

### 2.1. MCT2 rs3763980

**Figure 1:**
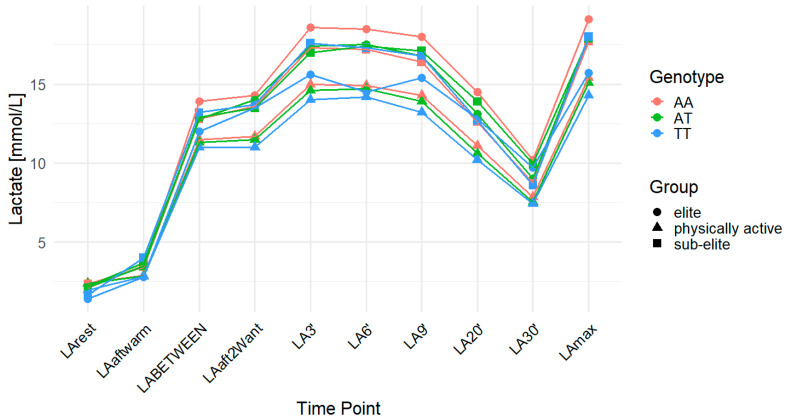
LA concentration over time across genotypes for the *MCT2* (monocarboxylate transporter 2) rs3763980 polymorphism. The data are presented as the mean for each time point. LA—lactate; LArest—LA concentration before the warm-up at rest; LAaftwarm—LA after warm-up; LABETWEEN—LA in the third minute of rest after the first but before the second Wingate test; LAaft2Want—LA immediately after the second Wingate test; LA3′—LA at 3′ min post-second Wingate; LA6′—LA at 6′ min post-second Wingate, LA9′—LA at 9′ min post-second Wingate, LA20′—LA at 20′ min post-second Wingate; LA30′—LA at 30′ min after the second Wingate test; LAmax—maximum LA concentration; AA—homozygote for the major allele (A); AT—heterozygote; TT—homozygote for the minor allele (T).

The *MCT2* rs3763980 variant showed significant associations with LA-related traits. The analysis revealed a significant association with LA concentration at rest (LArest) for all observations combined. In the codominant model (*p* = 0.03; FDR-adjusted *p* = 0.04), individuals with the AA genotype showed the highest LArest values (2.35 mmol/L), followed by AT (2.26 mmol/L) and TT (1.76 mmol/L). The recessive model also reached significance (*p* = 0.02; FDR-adjusted *p* = 0.04), indicating lower LArest in TT homozygotes compared to AA and AT combined. In elite athletes, LArest values differed between genotypes in a manner consistent with the overall group, but the differences were not statistically significant (*p* > 0.05 in all models). For ACC in elite athletes, associations were observed in the codominant (*p* = 0.04; FDR-adjusted *p* = 0.06) and dominant (*p* = 0.02; FDR-adjusted *p* = 0.06) models, but these did not remain significant after FDR correction. In the elite group, individuals with the AA genotype exhibited the highest ACC values (17.02 mmol/L), followed by AT (15.78 mmol/L) and TT (14.29 mmol/L). The effect sizes and post hoc test power estimates are presented in Table 3. Figure 1 provides a time-course visualization of LA concentrations across genotypes and training groups.

### 2.2. MCT2 rs995343

**Figure 2:**
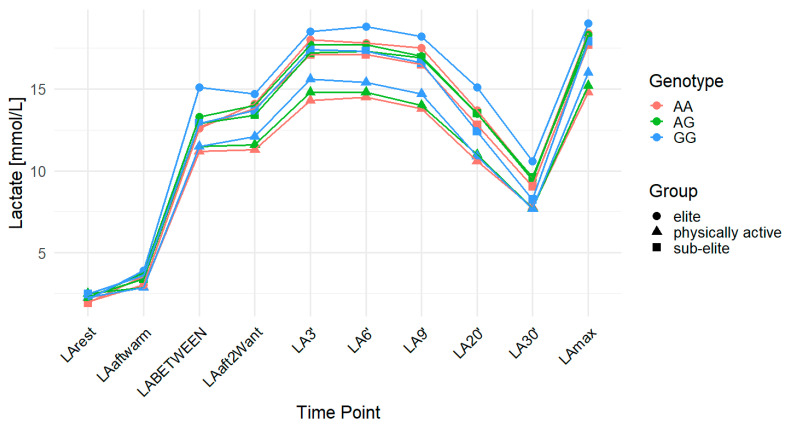
LA concentration over time across genotypes for the *MCT2* (monocarboxylate transporter 2) rs995343 polymorphism. The data are presented as the mean for each time point. LA—lactate; LArest—LA concentration before the warm-up at rest; LAaftwarm—LA after warm-up; LABETWEEN—LA in the third minute of rest after the first but before the second Wingate test; LAaft2Want—LA immediately after the second Wingate test; LA3′—LA at 3′ min post-second Wingate; LA6′—LA at 6′ min post-second Wingate, LA9′—LA at 9′ min post-second Wingate, LA20′—LA at 20′ min post-second Wingate; LA30′—LA at 30′ min after the second Wingate test; LAmax—maximum LA concentration; AA—homozygote for the major allele (A); AG—heterozygote; GG—homozygote for the minor allele (G).

The *MCT2* rs995343 variant was associated with LArest in the overall cohort. Higher LArest values were observed in G allele carriers, with significant associations in the codominant (*p* = 0.03; FDR-adjusted *p* = 0.04) and dominant (*p* = 0.01; FDR-adjusted *p* = 0.03) models (AA: 2.10 mmol/L, AG: 2.40 mmol/L, GG: 2.30 mmol/L). In the sub-elite group, an association was found in the dominant model (*p* = 0.04), but it did not remain significant after FDR correction (FDR-adjusted *p* = 0.12). In the physically active group, significant associations were found for LAmax in the codominant (*p* = 0.03; FDR-adjusted *p* = 0.04) and recessive (*p* = 0.02; FDR-adjusted *p* = 0.04) models (AA: 14.77 mmol/L, AG: 15.25 mmol/L, GG: 15.98 mmol/L). In the physically active group, ACC was also significantly associated with this variant in the codominant (*p* = 0.02; FDR-adjusted *p* = 0.03) and recessive (*p* = 0.01; FDR-adjusted *p* = 0.03) models (AA: 12.54 mmol/L, AG: 12.74 mmol/L, GG: 13.71 mmol/L). For DCC, the recessive model in the overall cohort showed a raw *p*-value of 0.02 but did not reach statistical significance after FDR correction (FDR-adjusted *p* = 0.06). In contrast, significant associations with DCC were found in the physically active group across all genetic models: codominant (*p* = 0.01; FDR-adjusted *p* = 0.02), dominant (*p* = 0.03; FDR-adjusted *p* = 0.03), and recessive (*p* = 0.01; FDR-adjusted *p* = 0.02) (AA: 7.00 mmol/L, AG: 7.55 mmol/L, GG: 8.31 mmol/L). The effect sizes and post hoc test power estimates are presented in Table 4. Figure 2 illustrates the time course of LA concentrations by genotype and training group.

### 2.3. MCT2 rs3763979

**Figure 3:**
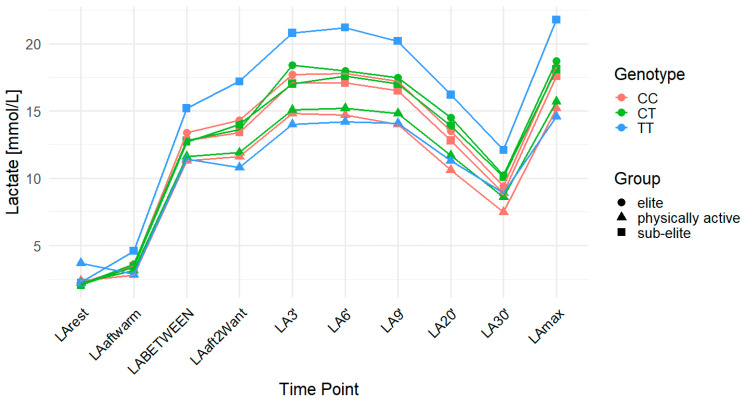
LA concentration over time across genotypes for the *MCT2* (monocarboxylate transporter 2) rs3763979 polymorphism. The data are presented as the means for each time point. LA—lactate; LArest—LA concentration before the warm-up at rest; LAaftwarm—LA after warm-up; LABETWEEN—LA in the third minute of rest after the first but before the second Wingate test; LAaft2Want—LA immediately after the second Wingate test; LA3′—LA at 3′ min post-second Wingate; LA6′—LA at 6′ min post-second Wingate, LA9′—LA at 9′ min post-second Wingate, LA20′—LA at 20′ min post-second Wingate; LA30′—LA at 30′ min after the second Wingate test; LAmax—maximum LA concentration; CC—homozygote for the major allele (C), CT—heterozygote, TT—homozygote for the minor allele (T).

The *MCT2* rs3763979 polymorphism was associated with multiple LA-related traits. Higher LArest values were observed in individuals with the TT genotype compared to C allele carriers, with significant associations in the recessive model for all observations combined (*p* = 0.003; FDR-adjusted *p* = 0.009; CC: 2.30 mmol/L, CT: 2.16 mmol/L, TT: 3.28 mmol/L) and in both the codominant (*p* = 0.003; FDR-adjusted *p* = 0.005) and recessive (*p* = 0.001; FDR-adjusted *p* = 0.003) models within the physically active group (CC: 2.37 mmol/L, CT: 2.24 mmol/L, TT: 3.69 mmol/L). This variant also influenced LA30′ with significant associations observed in the dominant model for the overall sample (*p* = 0.001; FDR-adjusted *p* = 0.003; CC: 8.13 mmol/L, CT: 9.33 mmol/L, TT: 9.84 mmol/L) and in the physically active group, where significant associations were found in the dominant (*p* = 0.01; FDR-adjusted *p* = 0.03) and codominant (*p* = 0.03; FDR-adjusted *p* = 0.04) models (CC: 7.46 mmol/L, CT: 8.59 mmol/L, TT: 8.92 mmol/L). LAmax was significantly associated with the rs3763979 variant in the sub-elite group under the recessive model (*p* = 0.04; FDR-adjusted *p* = 0.12), but this did not remain significant after FDR correction (CC: 17.64 mmol/L, CT: 17.97 mmol/L, TT: 21.78 mmol/L). A similar association was observed for ACC in the same group and model (*p* = 0.04; FDR-adjusted *p* = 0.12; CC: 15.41 mmol/L, CT: 15.91 mmol/L, TT: 19.53 mmol/L). For DCC, an association was observed in the dominant model for the overall cohort (*p* = 0.02; FDR-adjusted *p* = 0.06), with mean DCC values of 8.17 mmol/L for CC, 7.53 mmol/L for CT, and 6.84 mmol/L for TT. In the physically active group, associations were found in the codominant (*p* = 0.04; FDR-adjusted *p* = 0.06) and dominant (*p* = 0.03; FDR-adjusted *p* = 0.06) models (CC: 7.73 mmol/L, CT: 7.07 mmol/L, TT: 5.72 mmol/L). However, none of these associations remained statistically significant after FDR correction. TT genotype carriers consistently showed the lowest DCC values in both the overall cohort and the physically active group. Notably, the TT genotype was not present in the elite group (Table 5). Figure 3 provides a time-course of LA concentrations across genotypes and training groups.

### 2.4. MCT4 rs11323780

**Figure 4:**
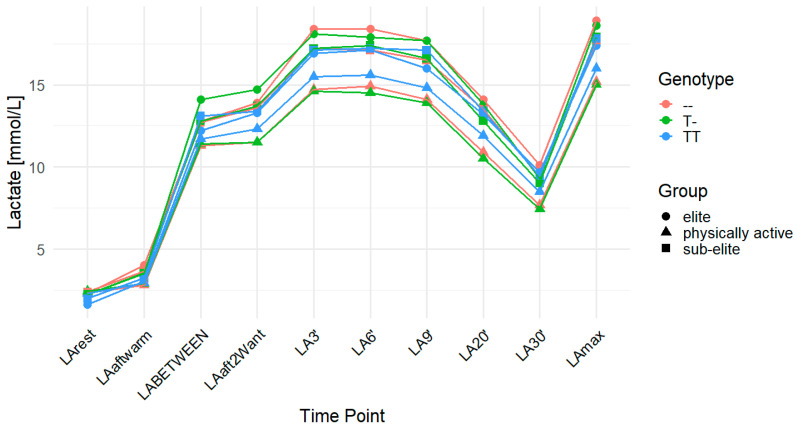
LA concentration over time across genotypes for the *MCT4* (monocarboxylate transporter 4) rs11323780 polymorphism. The data are presented as the means for each time point. LA—lactate; LArest—LA concentration before the warm-up at rest; LAaftwarm—LA after warm-up; LABETWEEN—LA in the third minute of rest after the first but before the second Wingate test; LAaft2Want—LA immediately after the second Wingate test; LA3′—LA at 3′ min post-second Wingate; LA6′—LA at 6′ min post-second Wingate, LA9′—LA at 9′ min post-second Wingate, LA20′—LA at 20′ min post-second Wingate; LA30′—LA at 30′ min after the second Wingate test; LAmax—maximum LA concentration; --—homozygote for the major allele; T-—heterozygote; TT—homozygote for the minor allele (T).

For LArest, the rs11323780 variant demonstrated an association in the elite group under the codominant (*p* = 0.04; FDR-adjusted *p* = 0.06) and recessive (*p* = 0.01; FDR-adjusted *p* = 0.03) models. However, only the result in the recessive model remained statistically significant after FDR correction. The mean LArest concentrations were higher in individuals with the T- (2.33 mmol/L) and -- (2.31 mmol/L) genotypes compared to TT homozygotes (1.62 mmol/L). In the overall cohort, LArest was associated with the recessive model (*p* = 0.02), but this association did not remain significant after FDR correction (FDR-adjusted *p* = 0.06; TT: 2.09 mmol/L, T-: 2.35 mmol/L, --: 2.35 mmol/L). In the physically active group, no statistically significant association was observed for LA30′ after FDR correction, although the recessive model showed a raw *p*-value of 0.04 (FDR-adjusted *p* = 0.12), with mean concentrations of 7.43 mmol/L for TT, 7.68 mmol/L for T-, and 8.49 mmol/L for -- genotypes. Similarly, LAmax in this group was not statistically significant after FDR correction (recessive model: *p* = 0.02; FDR-adjusted *p* = 0.06), with mean values of 15.00 mmol/L (TT), 15.22 mmol/L (T-), and 16.00 mmol/L (--). For ACC, the association in the overall cohort under the recessive model showed a raw *p*-value of 0.02 but did not reach statistical significance after FDR correction (FDR-adjusted *p* = 0.06; TT: 14.77 mmol/L, T-: 13.92 mmol/L, --: 14.01 mmol/L). In the physically active group, ACC was significantly associated with rs11323780 in both the codominant (*p* = 0.02; FDR-adjusted *p* = 0.03) and recessive (*p* = 0.01; FDR-adjusted *p* = 0.03) models, with increasing concentrations from TT (12.56 mmol/L) to T- (12.88 mmol/L) to -- (13.72 mmol/L). No statistically significant associations with DCC were observed in any group after FDR correction (Table 6). Figure 4 illustrates the changes in LA concentrations over time among different genotypes and training groups.

### 2.5. Correlation Analysis

The analysis of correlations between LA-related variables revealed several significant relationships. LABETWEEN and LAaft2WAnt were highly correlated (*r* = 0.78), showing strong associations with LA3′ (*r* = 0.80 and *r* = 0.75, respectively). Similarly, LA3′ demonstrated strong positive correlations with LA6′ (*r* = 0.92), LA9′ (*r* = 0.86), LA20′ (*r* = 0.73), and LA30′ (*r* = 0.60). A powerful relationship was observed between LA3′ and LAmax (*r* = 0.95), further emphasizing the importance of early LA kinetics in determining maximal LA accumulation.

LA6′ was strongly correlated with LA9′ (*r* = 0.93), LA20′ (*r* = 0.81), LA30′ (*r* = 0.69), and LAmax (*r* = 0.97). The correlation between LA9′ and LA20′ was also high (*r* = 0.85), as was the association between LA9′ and LA30′ (*r* = 0.75). These findings suggest that LA clearance over time follows a predictable pattern, with values at different time points strongly interrelated.

ACC was correlated highly with LA3′ (*r* = 0.90), LA6′ (*r* = 0.92), and LA9′ (*r* = 0.91), further confirming that LA dynamics play a key role in short-term performance. In contrast, DCC exhibited weaker correlations with LA parameters, with the highest associations observed with LA3′ (*r* = 0.39) and LAaft2WAnt (*r* = 0.32), while showing negative correlations with LA20′ (*r* = −0.22). These results indicate that while LA accumulation and clearance are closely linked to performance, different LA variables may have distinct influences depending on the recovery phase and the effort exerted (Table 7).

## 3. Discussion

Prior studies have investigated the roles of MCT4 [28,29] in LA accumulation and removal during and after exercise in humans. However, those studies focus on protein expression, content, and physiological function, not on genetic polymorphisms of *MCT4*. Our 2025 cross-sectional study involving 337 male athletes from Poland and the Czech Republic appears to be the only published study directly investigating the relationship between *MCT2* and *MCT4* gene polymorphisms and LA accumulation/removal in men during high-intensity exercise [21]. However, this study included novel haplotype analysis and intergenic interactions without examining the independent effects of *MCT2* and *MCT4* gene variants [21]. In the present study, we specifically investigated the individual significance of these polymorphisms on LA utilization. Using SNP genotyping, we investigated the genetic predisposition for efficient LA utilization in elite, sub-elite sprinters, and physically active men (controls).

In this study, LA accumulation refers to the build-up of LA in the bloodstream, a phenomenon observed particularly during exercise when the intensity surpasses the body’s capacity to remove it [27]. As in our previous study [21], blood LA concentrations were assessed in the current work. However, one might question the reliability of this approach and propose that muscle LA could serve as a more precise marker. The buildup of LA results from the enhanced conversion of pyruvate to LA, largely influenced by changes in the intramuscular redox environment. Additionally, the utilization of excess LA is contingent upon its distribution through blood flow to other muscles, the heart, and the liver [30]. As such, LA concentrations in muscle tissues provide insight into blood LA concentrations [31]. Furthermore, while the accumulation of LA—whether in muscle or blood—signals an increase in proton release and a potential drop in pH levels within cells and blood, it is acknowledged in standard medical practice that a heightened concentration of blood LA is a recognized indicator of oxygen deficiency [32].

Our results revealed that specific polymorphisms in the *MCT2* and *MCT4* genes significantly influence blood LA kinetics following high-intensity exercise, underscoring the genetic component of LA metabolism in athletes. LA concentrations were measured at various time points. Additionally, ACC, DCC, and LAmax were calculated. ACC quantifies the extent to which LA concentration increases during exercise. A greater ACC indicates an enhanced ability to produce or tolerate LA, which may be advantageous in short, intense efforts where anaerobic metabolism predominates. However, high accumulation can also reflect impaired LA clearance or transport. DCC refers to the body’s ability to metabolize and eliminate LA from the bloodstream post-exercise.

The *MCT2* rs3763980 polymorphism demonstrated significant associations with LArest and ACC. For LArest, individuals with the TT genotype had the lowest concentrations, and this association remained statistically significant in the codominant (FDR-adjusted *p* = 0.04) and recessive (FDR-adjusted *p* = 0.04) models when considering all participants combined. Although subgroup analyses (elite, sub-elite, and physically active) showed the same genotype ranking (AA > AT > TT), these differences were not statistically significant within any group. Differences in LA values at later time points such as LA30′ followed a similar genotype pattern but did not reach statistical significance. Given that MCT2 exhibits high affinity for LA transport in oxidative tissues [6,7], these findings may reflect genotype-dependent efficiency in basal LA metabolism or transport. Although the codominant (*p* = 0.04; FDR-adjusted *p* = 0.06) and dominant (*p* = 0.02; FDR-adjusted *p* = 0.06) models suggested differences in ACC in the elite group, these associations did not remain statistically significant after correction for multiple comparisons. Therefore, no firm conclusions regarding genotype-specific effects on ACC can be drawn. No significant associations were observed for DCC in any group or genetic model. These findings may suggest a potential role of rs3763980 in influencing basal LA concentrations (LArest), while no statistically significant effects were observed for LA clearance during recovery (DCC).

The *MCT2* rs995343 variant demonstrated significant associations with LArest in the overall cohort, but not in subgroup analyses. In the full sample, codominant (*p* = 0.03; FDR-adjusted *p* = 0.04) and dominant (*p* = 0.01; FDR-adjusted *p* = 0.03) models showed that carriers of the G allele (AG and GG) had higher LArest values compared to AA homozygotes, with AG individuals presenting the highest mean. In the sub-elite group, the dominant model showed a raw *p*-value of 0.04, but the result did not remain significant after FDR correction (FDR-adjusted *p* = 0.12). This elevation in resting LA concentrations among G allele carriers could reflect underlying physiological differences in LA transport. The observed differences in resting LA may be partially explained by *MCT2* kinetics, as its high affinity facilitates LA uptake under low-concentration [33] conditions typical of rest [15]. Given that MCT2 is a high-affinity LA transporter, variations at rs995343 may also influence cellular LA uptake and systemic concentration at rest.

In the physically active group, significant associations were found for LAmax in the codominant (*p* = 0.03; FDR-adjusted *p* = 0.04) and recessive (*p* = 0.02; FDR-adjusted *p* = 0.04) models, and for ACC in the codominant (*p* = 0.02; FDR-adjusted *p* = 0.03) and recessive (*p* = 0.01; FDR-adjusted *p* = 0.03) models. Individuals with the GG genotype had the highest mean LAmax and ACC values. These results suggest that GG genotype carriers may have a greater capacity for LA accumulation during high-intensity anaerobic exercise, possibly reflecting a more glycolytic phenotype. This interpretation aligns with previous research linking *MCT* polymorphisms to anaerobic metabolism and sprint performance [14,21].

For DCC, the recessive model in the overall cohort showed a raw *p*-value of 0.02 but did not remain statistically significant after FDR correction (FDR-adjusted *p* = 0.06). In the physically active group, however, significant associations were found across all models (codominant, dominant, and recessive), with GG homozygotes exhibiting the highest DCC concentrations. These findings may indicate increased post-exercise LA clearance in GG genotype carriers, potentially reflecting enhanced MCT2-mediated LA transport or mitochondrial oxidation. MCT2 is involved in LA influx into oxidative muscle fibers, which can be used as a substrate for aerobic metabolism [34]. Therefore, the *MCT2* rs995343 variant may influence both LA accumulation and clearance dynamics, particularly in physically active individuals, with GG homozygotes showing the most pronounced effects.

The analysis of *MCT2* rs3763979 revealed significant associations with LArest and LA30′, particularly in the overall and physically active cohorts. For LA30′, TT genotype carriers also demonstrated the highest post-exercise concentrations. This association remained statistically significant in the overall group (dominant model: FDR-adjusted *p* = 0.003) and the physically active group under both the dominant (FDR-adjusted *p* = 0.03) and codominant (FDR-adjusted *p* = 0.04) models. These results suggest that the rs3763979 variant may influence early LA accumulation after intense effort. For LArest, individuals with the TT genotype exhibited the highest concentrations, and this difference remained statistically significant after FDR correction in both the overall cohort (recessive model: FDR-adjusted *p* = 0.009) and the physically active group (codominant: FDR-adjusted *p* = 0.005; recessive: FDR-adjusted *p* = 0.003). These findings suggest that the TT genotype may be associated with elevated resting LA concentrations. MCT2, a high-affinity LA transporter [6], is abundantly expressed in oxidative muscle fibers [35,36], where it facilitates LA influx under resting conditions [6,37]. Moreover, MCT2 participates in the mitochondrial LA oxidation complex (mLOC), which includes mitochondrial LA dehydrogenase and cytochrome oxidase. This complex enables LA oxidation within mitochondria, contributing to efficient energy production in oxidative muscle fibers [38]. Therefore, the elevated resting LA concentrations observed in TT genotype carriers may reflect altered transporter function, possibly involving reduced LA influx into oxidative tissues or impaired mitochondrial utilization, leading to higher systemic LA accumulation at rest. In contrast, no statistically significant associations were found for LAmax or ACC after FDR correction. Interestingly, the TT genotype was not present among elite athletes in our cohort (discussed further in the Limitations section), which could hypothetically suggest a selection against this genotype at the highest levels of performance. However, this interpretation should be approached with caution. Overall, the findings support the role of the *MCT2* rs3763979 variant in modulating resting and post-exercise LA concentrations, particularly in physically active individuals. However, no firm conclusions can be drawn regarding its effect on maximal LA production or clearance during recovery.

The *MCT4* rs11323780 polymorphism showed associations with several LA-related traits, although only selected results remained statistically significant after FDR correction. For LArest, a statistically significant association was found in the elite athlete group under the recessive model (FDR-adjusted *p* = 0.03), with TT genotype carriers exhibiting lower concentrations compared to heterozygotes and homozygous variant carriers (T- and --). Although the codominant model also yielded a raw *p*-value of 0.04 in this group, it did not remain significant after correction (FDR-adjusted *p* = 0.06). A similar association was observed in the overall cohort under the recessive model (raw *p* = 0.02), but this result also did not remain significant after FDR correction (FDR-adjusted *p* = 0.06). These findings cautiously suggest that the TT genotype may be linked to lower resting LA concentrations in certain groups, but further confirmation is needed. This observation aligns with the known physiological role of MCT4. MCT4, primarily expressed in fast-twitch glycolytic muscle fibers, is a low-affinity, high-capacity LA transporter that plays a key role in LA efflux during exercise [39]. Although the functional effect of rs11323780 is not fully established, a variant at this locus may influence MCT4 transporter kinetics or expression, potentially contributing to more efficient resting LA handling. Rs11323780 could influence transporter efficiency, leading to altered LA handling at rest. However, this remains speculative, as the direct functional impact of this SNP on MCT4 expression or activity has not been experimentally verified. The association between the TT genotype and reduced LArest may imply a more efficient resting-state clearance or lower LA production, which could benefit elite athletes’ performance and recovery. In the physically active group, no statistically significant associations were found for LA30′ or LAmax after FDR correction, although both traits showed raw *p*-values below 0.05 in the recessive model (*p* = 0.04 and *p* = 0.02, respectively; FDR-adjusted *p* = 0.12 and 0.06). In contrast, ACC in the physically active group remained statistically significant after FDR adjustment under both the codominant (FDR-adjusted *p* = 0.03) and recessive (FDR-adjusted *p* = 0.03) models. Individuals with the -- genotype had the highest mean ACC values, followed by T- and TT. This may indicate that the rs11323780 variant modulates LA accumulation capacity during high-intensity exercise, at least in this subgroup. These findings suggest that individuals with the TT genotype may accumulate less LA during and after high-intensity exercise, potentially reflecting a genotype-related difference in LA handling. However, this observation requires further validation in mechanistic and longitudinal studies. No statistically significant associations were observed for DCC in any group or model after FDR correction. Therefore, rs11323780 does not appear to influence post-exercise LA clearance in a consistent or robust manner. Overall, these findings suggest that the MCT4 rs11323780 polymorphism may contribute to interindividual differences in LA accumulation, particularly under high-intensity conditions in physically active individuals. These findings suggest that *MCT4* rs11323780 may influence LA accumulation during high-intensity exercise, possibly via altered transport capacity, though no consistent effect on clearance was observed. While the T allele appears to influence LA concentrations during and immediately after high-intensity effort, it does not seem to affect the delayed phase of LA removal. This pattern may suggest that the *MCT4* rs11323780 polymorphism primarily affects LA handling during exertion, rather than recovery, possibly due to differences in transport activity under peak metabolic stress. However, no firm conclusions can be drawn regarding its role in LA clearance during recovery.

The correlation analysis of LA parameters revealed a physiologically coherent pattern across exercise and recovery phases. LABETWEEN and LAaft2WAnt were strongly correlated with each other (*r* = 0.78), and both also demonstrated strong associations with LA3′ (*r* = 0.80 and *r* = 0.75, respectively), suggesting that early post-exercise LA concentrations reflect accumulation during exercise and the immediate recovery period. LA3′, as a key early recovery marker, demonstrated correlations with subsequent time points, including LA6′ (*r* = 0.92), LA9′ (*r* = 0.86), LA20′ (*r* = 0.73), and LA30′ (*r* = 0.60), indicating a predictable clearance trajectory across time. LA6′ and LA9′ were likewise strongly associated with each other (*r* = 0.93) and with later time points, such as LA20′ (*r* = 0.81) and LA30′ (*r* = 0.69), reinforcing the consistency of LA removal dynamics during recovery. LAmax, representing the peak value reached during or immediately after exertion, also correlated strongly with early post-exercise values such as LA3′ (*r* = 0.95), LA6′ (*r* = 0.97), and LA9′ (*r* = 0.91). These strong correlations reflect the internal consistency of LA and suggest that early-phase markers can reliably predict peak accumulation. ACC also showed high correlations with LA3′ (*r* = 0.90), LA6′ (*r* = 0.92), and LA9′ (*r* = 0.91), but this likely reflects mathematical dependence, as ACC is derived from the same data as LAmax. Therefore, while the relationships are numerically strong, they do not necessarily imply an independent physiological mechanism. In contrast, DCC showed only weak-to-moderate correlations with other LA variables. It was weakly positively correlated with LA3′ (*r* = 0.39) and LAaft2WAnt (*r* = 0.32), and negatively with LA20′ (*r* = –0.22) and LA30′ (*r* = –0.45). These weaker and more variable associations suggest that DCC reflects a partially independent physiological process, possibly related to individual differences in buffering capacity, oxidative metabolism, or recovery kinetics.

Overall, the results highlight the central role of early post-exercise LA values (especially LA3′ and LA6′) in describing the kinetics of LA accumulation and clearance. In contrast, DCC may be more effective in capturing delayed or individual-specific aspects of recovery that are not as closely associated with peak LA dynamics. Therefore, the results indicate that LA accumulation is interconnected across time points, whereas clearance metrics, such as DCC, may reflect more independent recovery processes, potentially influenced by other physiological mechanisms.

There are no publications linking *MCT2* SNPs (rs3763980, rs995343, rs3763979) and *MCT4* (rs11323780) with LA utilization. *MCT2* rs3763980 is linked to methotrexate efficacy [25,26], while rs995343 lung [23] and colorectal cancer [24], and there are no publications for rs3763979. To our knowledge, this is the first study to report the independent effects of *MCT2* and *MCT4* gene variants on LA-related traits. Our findings provide novel insights into the genetic determinants of LA kinetics, encompassing peak LA concentration (LAmax), early and late phase clearance (e.g., LA3′, LA20′, LA30′), total LA accumulation during exercise (ACC), and overall LA removal capacity during recovery (DCC). This study thus contributes valuable new genetic evidence to exercise physiology, metabolic adaptation, and molecular biology.

### Limitations

The lack of literature makes it challenging to refer to any existing data. At the same time, our results contribute to the existing knowledge, as our research topic is not well-studied, and our work is exploring new territory. On the contrary, a limitation of the current study is the need for more insights into Polish and Czech females. The significance of incorporating diverse groups in sports science research is recognized, as gender variances can greatly influence various results. The choice to concentrate on a male-only group in this research was made due to the scale of the intervention and the limited sample size of female participants available at the time. We continue recruiting female participants. However, obtaining consent from women for this study has proven more challenging. Nevertheless, we acknowledge the importance of involving women in upcoming studies to better comprehend the observed effects. Another noteworthy limitation of this study is the relatively small sample size, particularly for the elite athlete group (*n* = 42). Unfortunately, recruiting elite athletes poses significant logistical and practical challenges due to their limited availability, demanding training schedules, and highly selective nature. The 42 elite sprinters included in our study represent a specific and rigorously selected population, which hinders efforts to expand this sample meaningfully. Additionally, the absence of the TT genotype for rs3763979 in this subgroup further limits the possibility of genotype-specific interpretation. Future studies with larger and more diverse elite athlete cohorts are warranted to strengthen the robustness and generalizability of our findings. Next, the credibility and reliability of the results are contingent on replication studies. The observed variations in LA utilization associated with specific gene variants suggest that the genetic landscape and cellular context are complex. The interplay of genetic, environmental, and regulatory factors contributes to the overall phenotype, and understanding these nuances is essential for unraveling the complete picture of LA metabolism in different individuals. The study is limited by its lack of examination of the complex interactions among genetic variations, cultural influences, epigenetic modifications, and environmental factors. Cultural aspects, such as dietary practices, physical activity patterns, and training methodologies, can modulate gene expression and metabolic pathways, affecting LA kinetics and overall athletic performance. Future research should focus on delineating these interactions more comprehensively. It should be noted that the present study relies solely on genotype–phenotype associations and does not include molecular-level functional assays such as mRNA or protein quantification or direct measurements of transporter activity. We acknowledge that such analyses would provide stronger mechanistic insights into the observed associations. Nonetheless, we sought to address this limitation indirectly by incorporating lactate kinetics parameters derived from repeated Wingate tests, i.e., LA30′, LAmax, ACC, and DCC. While these do not replace molecular validation, they represent relevant performance-based phenotypes that reflect the physiological consequences of genetic variation. Future studies should integrate biochemical techniques, such as Enzyme-Linked Immunosorbent Assay (ELISA) or mRNA quantification, to examine protein-level expression and function concerning genotype. Additionally, incorporating epigenetic analyses could provide further insights into how environmental and cultural factors influence gene regulation and athletic performance. Future studies could also involve longitudinal designs that track athletes to assess how changing dietary habits or training techniques affect their physiological responses and athletic outcomes, holistically integrating genetic and cultural dimensions. Finally, the cross-sectional approach can demonstrate associations but cannot determine causality between genetic variations and LA kinetics.

## 4. Materials and Methods

### 4.1. Study Design

**Figure 5:**
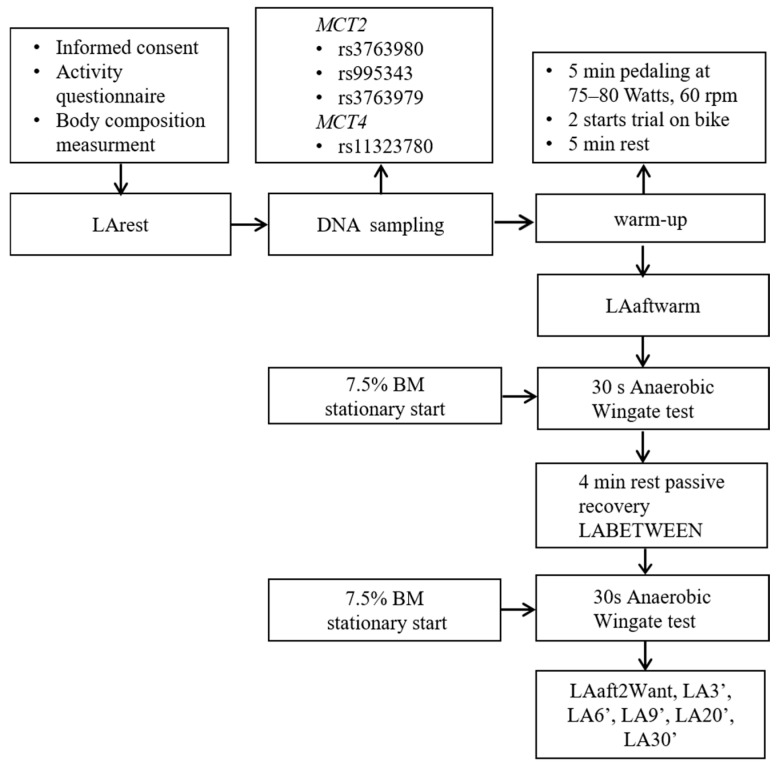
Schematic diagram of the research experiment. LA—lactate; LArest—LA concentration before the warm-up at rest; LAaftwarm—LA after warm-up; LABETWEEN—LA in the third minute of rest after the first but before the second Wingate test; LAaft2Want—LA immediately after the second Wingate test; LA3′—LA at 3′ min post-second Wingate; LA6′—LA at 6′ min post-second Wingate, LA9′—LA at 9′ min post-second Wingate, LA20′—LA at 20′ min post-second Wingate; LA30′—LA at 30′ min after the second Wingate test; *MCT2*—monocarboxylate transporter 2; *MCT4*—monocarboxylate transporter 4; BM—body mass.

This cross-sectional control study explored the correlation between genetic factors and the acute physiological responses to high-intensity glycolytic exertion, explicitly focusing on LA production and clearance post-exercise. Individuals were chosen based on health and performance standards, ensuring their fundamental ability to undergo two intermittent anaerobic all-out tests (Figure 5). First, the participants provided informed consent and filled in a physical activity questionnaire. DNA samples were taken at rest before body composition measurements and warm-up. Subsequently, two intermittent all-out Wingate tests were implemented. LA concentrations were assessed before the warm-up at rest (LArest), after warm-up (LAaftwarm), in the third minute of rest after the first but before the second Wingate test (LABETWEEN), immediately after the second Wingate (LAaft2Want), and at 3′, 6′, 9′, 20′ and 30′ minutes (min) after the second Wingate. The two intermittent all-out Wingate tests were conducted at the Physiological Laboratory of the Department of Biomedical Sciences at the University of Physical Education in Warsaw.

### 4.2. Participants

This research included a cohort of 337 male athletes aged 16 to 29 years. The sample consisted of 42 elite athletes (28 from the Czech Republic and 14 from Poland), 103 sub-elite athletes (with 40 participants from Poland and 63 from the Czech Republic), and 192 physically active individuals from Poland who were not engaged in competitive speed or strength sports (control group). All participants provided informed consent to participate in the study. The following were adopted as inclusion criteria for the elite and sub-elite sample: needed to achieve a running distance of 400 m in less than 50 s (elite) and more (sub-elite) during the current season, participate in at least four workouts per week, and attend 80% of club workouts to be included in the study. The participants who had engaged in high-intensity sports or workouts within the last 72 h, sustained an injury in the past three months, and did not have clearance from a sports doctor were not eligible for inclusion. The elite athlete group (*n* = 42) had an average age of 20.48 ± 2.80 years, weight of 73.65 ± 14.72 kg, and height of 182.87 ± 6.42 cm. In contrast, the sub-elite group (*n* = 145) had an average age of 19.79 ± 2.80 years, 72.92 ± 9.24 kg, and 182.67 ± 5.67 cm height. The physically active group (*n* = 192) had an average age of 20.94 ± 1.90 years, weight of 77.44 ± 9.61 kg, and height of 180.73 ± 6.50 cm. Before commencing the study, all individuals were provided with a document containing information about the study’s specifics, objectives, processes, possible risks, and the advantages of their involvement. They also filled out a survey form. This questionnaire collected a wide range of information, including demographic and anthropometric data (age, sex, height, weight, ethnicity, nationality), sports background (primary sport discipline, training experience, weekly training volume, competition history, personal bests in 200 m and 400 m), and recent training loads. It also included health-related questions, such as recent injuries, medical clearance for physical activity, chronic conditions, and a history of COVID-19. The information was used to confirm eligibility and describe the characteristics of the study population.

### 4.3. Intermittent All-Out Wingate Tests

Athletes performed a 5-min warm-up on a cycling ergometer (Monark 894 E peak bike, Sweden) at a 1 Watt/kg intensity with a cadence of 60 revolutions per minute (rpms), incorporating two accelerations lasting 3–5 s, before undertaking the Wingate test. Following the warm-up, the participants rested for 5 min. The Wingate test was chosen because prior studies had demonstrated a genetic correlation between elite athletes’ genetic makeup and Wingate outputs [40]. Two successive all-out Wingate tests were performed as part of the protocol to induce a maximal glycolytic response. During the test, individuals were kept seated, and resistance was supplied at a rate of 7.5% of their body mass. The Wingate test was initiated from a standstill [41]. Following the load setup (7.5% body mass), the subject’s feet were attached to the pedals. After a five-second countdown, they began the test with all their might. The power was measured when the subjects began the test. For 30 s, the subjects pedaled as quickly as they could. Following the test, the participants underwent a 4-min passive rest [42] before completing a follow-up test following the identical protocol (Figure 5). After completing the second Wingate test, the participants took a 4-min active rest on a cycling ergometer, pedaling at an intensity of approximately 50 revolutions per minute.

### 4.4. Blood LA Measurement

Capillary blood samples were obtained from the fingertip nine times: before the warm-up at rest (LArest), following the warm-up (LAaftwarm), during the third minute of rest after the first but before the second Wingate test (LABETWEEN), immediately after the second Wingate (LAaft2Want), and during recovery (LA3′, LA6′, LA9′, LA20′, and LA30′) (Figure 5). The samples were drawn into end-to-end capillaries treated with sodium heparin. The Biosen device, which measures LA within the range of 0.5–40 mmol/L (5–360 mg/dl), was used to calculate the LA concentration (Biosen C-Line Lactate analyzer, EKF Diagnostics, Barleben, Germany).

### 4.5. DNA Sampling and Isolation

Participants’ biological material was obtained by collecting scrapings from the inner cheek utilizing two Copan FLOQSwab samples (Interpath, Melbourne, Australia) following the manufacturer’s standard protocol. Genomic DNA from the epithelial cell was isolated using commercial High Pure PCR kits (Roche Diagnostics, Mannheim, Germany) according to the manufacturer’s instructions. The quality and quantity of DNA samples were verified using a spectroscopic photometer (NanoPhotometer NP80, Munich, Germany), and, subsequently, the samples were stored at −20 °C for subsequent analysis.

### 4.6. Genotyping Analyses

Genotyping for all samples was duplicated using the Real-Time PCR system QuantStudio1 (Applied Biosystems, Waltham, USA). TaqManTM probes were employed for the four SNPs of the *MCT2* gene polymorphisms rs3763980 (C_25982884_10), rs995343 (C_7523041_10), rs3763979 (C_395113_10), and *MCT4* gene rs11323780 (C_175816943_10) (Applied Biosystems, Waltham, USA), utilizing starters and fluorescently labeled (VIC and FAM) MGBTM allele detection probes. The SNPs rs3763980, rs995343, and rs3763979 within the *MCT2* gene were selected due to the absence of existing literature linking these variants with LA production and clearance following high-intensity exercise. Similarly, few studies have examined the link between *MCT4* rs11323780 and LA dynamics under these conditions. Genotyping followed the manufacturer’s protocol using TaqPathTM ProAmpTM Master Mix (Applied Biosystems, USA). PCR reaction components (10 μL) were prepared by combining 5.0 μL of the master mix, 0.5 μL of the TaqMan SNP Genotyping Assay, and 4.5 μL of DNA. PCR ED reaction components (10 μL) included 5.0 μL of the master mix, 0.5 μL of the TaqMan SNP Genotyping Assay, and 4.5 μL of nuclease-free water. Reaction conditions included an initial denaturation at 95 °C for 5 min, followed by 40 cycles of denaturing at 95 °C for 15 s, starter hybridization and extension at 60 °C for 1 min, and a final extension at 60 °C for 30 s. The amplified products were analyzed using QuantStudio 1 Real-Time PCR Instrument’s Design and Analysis Software version 1.5.1. Subsequently, the samples were scrutinized and stored in the Genetic Laboratory of the Department of Biomedical Sciences at Jozef Piłsudski University of Physical Education in Warsaw.

### 4.7. Statistical Analyses

All statistical analyses were conducted using R software, version 4.4.3. Statistical significance was determined at the <0.05 standard level. The normality of the datasets was assessed using the Shapiro–Wilk test. A *t*-test was applied to compare two different groups. When analyzing more than two groups, a one-way Analysis of Variance (ANOVA) was conducted to determine whether significant differences existed. If ANOVA results were statistically significant, post hoc pairwise comparisons were performed with appropriate adjustments for multiple testing. A two-way ANOVA examined potential interactions between genotype, LA mean values, and distribution to sub-elite and physically active groups. This approach enabled the assessment of the main effects and any interaction effects between these factors. Furthermore, the effect of the genotype on LA parameters was evaluated using three genetic models: codominant (A/A vs. A/a vs. a/a), dominant (A/A vs. A/a + a/a), and recessive (A/A + A/a vs. a/a). For post hoc comparisons, Fisher’s Least Significant Difference (LSD) test was employed. The analysis of a single locus was performed based on four genetic models: codominant, dominant, recessive, and over-dominant. These models were constructed concerning the minor allele. The odds ratio (OR) was employed to evaluate whether a specific genotype could be more relevant among sub-elites. The model’s fit to the data was assessed using the Akaike Information Criterion (AIC). However, the results are not shown as none of the models demonstrated statistical significance. The Hardy–Weinberg equilibrium (HWE) was tested to verify whether the observed genotype distributions conformed to expected frequencies within the studied population. Additionally, effect sizes (η^2^, d) along with their confidence intervals (CIs) were calculated, and post hoc statistical power was estimated. A false discovery rate (FDR) correction was applied to control for multiple comparisons.

## 5. Conclusions

This study demonstrated that genetic polymorphisms in *MCT2* and *MCT4* contribute to inter-individual variability in LA metabolism among male athletes. Among the analyzed variants, *MCT2* rs995343 exhibited the broadest relevance, with significant associations observed for LArest, LAmax, ACC, and DCC in physically active individuals. MCT2 rs3763979 was linked to both LArest and post-exercise LA (LA30′) in the overall and physically active cohorts, whereas *MCT2* rs3763980 was associated solely with LArest in the overall group. *MCT4* rs11323780 demonstrated group-specific associations with LArest in elite athletes and ACC in physically active individuals. These findings suggest that *MCT* gene variants may contribute to inter-individual differences in anaerobic performance and recovery. While such associations could offer future insights into talent identification or training personalization, these implications remain hypothetical and require confirmation through longitudinal and mechanistic studies. While the associations are interesting, the underlying biological mechanisms remain to be elucidated in functional studies. Future studies should validate these associations in larger, diverse cohorts and explore the mechanistic pathways linking genotype to LA kinetics. These findings support continued research into the genetic basis of LA kinetics, with future work needed to establish causal mechanisms and practical applications.

## Figures and Tables

**Table 1:** Summary of sample size, age, height, and body mass in the study groups.

Group	*n*	Age (Years)	Weight (kg)	Height (cm)
Elite	42	20.48 ± 2.80	73.65 ± 14.72	182.87 ± 6.42
Sub-elite	145	19.79 ± 2.80	72.92 ± 9.24	182.67 ± 5.67
Physically active	192	20.94 ± 1.90	77.44 ± 9.61	180.73 ± 6.50

The data are presented as mean ± standard deviation (SD).

**Table 2:** Minor allele frequency (MAF) and *p*-values from Hardy–Weinberg equilibrium (HWE) tests for *MCT2* (rs3763980, rs995343, rs3763979) and *MCT4* (rs11323780) polymorphisms in elite, sub-elite, and physically active groups.

	MAF (%)	HWE *p*-Value All	HWE *p*-Value Elite	HWE *p*-Value Sub-Elite	HWE *p*-Value Physically Active
*MCT2* (rs3763980)	allele T (24.18)	0.38	0.45	0.62	0.40
*MCT2* (rs995343)	allele G (43.77)	0.45	0.52	0.22	0.88
*MCT2* (rs3763979)	allele T (12.46)	0.22	1.00	0.66	0.19
*MCT4* (rs11323780)	allele T (47.33)	1.00	0.76	0.69	0.66

**Table 3:** Blood LA concentrations (mmol/L) in all, elite, sub-elite, and physically active individuals at rest, after exercise, and during recovery across genotypes in the *MCT2* rs3763980 polymorphism, analyzed using codominant, dominant, and recessive allele models, along with their effects (η^2^, d) with 95% CI and post hoc power.

		Genotype			Models			Effect (η^2^, d); (95% CI); Post Hoc Power		
		AA	AT	TT	Codominant	Dominant	Recessive	Codominant	Dominant	**Recessive**
					AA vs. AT vs. TT	AA vs. AT + TT	AA + AT vs. TT	AA vs. AT vs. TT	AA vs. AT + TT	**AA + AT vs. TT**
LArest	All	2.35	2.26	1.76	0.03/0.04 *	0.07	0.02/0.04 *	0.02; (0.00–0.10)	0.16; (−0.05–0.38)	0.63; (0.11–1.15)
	Elite	2.08	2.21	1.4	0.31	0.78	0.16	0.06; (0.00–0.36)	−0.09; (−0.69–0.52)	1.05; (−0.40–2.48)
	Sub-elite	2.33	2.11	1.65	0.16	0.13	0.13	0.04; (0.00–0.22)	0.30; (−0.09–0.69)	0.64; (−0.19–1.47)
	Physically active	2.41	2.37	1.96	0.43	0.54	0.21	0.009; (0.00–0.09)	0.09; (−0.20–0.38)	0.48; (−0.27–1.24)
LA30′	All	8.34	8.56	8.2	0.99	0.88	1	0.002; (0.00–0.03)	−0.06; (−0.28–0.15)	0.08; (−0.44–0.60)
	Elite	10.21	9.01	9.72	0.34	0.15	0.95	0.05; (0.00–0.35)	0.45; (−0.17–1.06)	−0.04; (−1.46–1.38)
	Sub-elite	8.72	9.96	8.6	0.13	0.08	0.6	0.04; (0.00–0.23)	−0.35; (−0.73–0.05)	0.22; (−0.61–1.05)
	Physically active	7.84	7.53	7.43	0.71	0.41	0.77	0.004; (0.00–0.06)	0.12; (−0.17–0.41)	0.11; (−0.64–0.87)
LAmax	All	16.47	16.42	15.94	0.68	0.46	0.51	0.002; (0.00–0.03)	0.03; (−0.18–0.25)	0.19; (−0.33–0.70)
	Elite	19.1	17.99	15.69	0.06	0.06	0.08	0.14; (0.00–0.49)	0.61; (−0.01–1.23)	1.31; (−0.15–2.75)
	Sub-elite	17.73	17.85	17.97	0.97	0.81	0.87	0.001; (0.00–0.01)	−0.05; (−0.43–0.34)	−0.07 (−0.89–0.76)
	Physically active	15.44	15.06	14.27	0.26	0.17	0.23	0.01; (0.00–0.11)	0.20; (−0.09–0.49)	0.46; (−0.29–1.22)
ACC	All	14.12	14.16	14.18	0.98	0.87	0.92	0; (0.00–0.00)	−0.02; (−0.23–0.20)	−0.02; (−0.53–0.50)
	Elite	17.02	15.78	14.29	0.04/0.06 *	0.02/0.06 *	0.14	0.2; (0.00–0.52); 0.8	0.8; (0.12–1.38); 0.7	1.10; (−0.34–2.54)
	Sub-elite	15.4	15.73	16.32	0.66	0.45	0.5	0.008; (0.00–0.11)	−0.15; (−0.54–0.24)	−0.29; (−1.11–0.54)
	Physically active	13.03	12.7	12.31	0.48	0.26	0.49	0.008; (0.00–0.08)	0.17; (−0.12–0.46)	0.27; (−0.49–1.02)
DCC	All	8.13	7.87	7.74	0.51	0.3	0.45	0.004; (0.00–0.05)	0.12; (−0.09–0.34)	0.13; (−0.39–0.64)
	Elite	8.89	8.98	5.97	0.24	0.81	0.09	0.07 (0.00–0.39)	0.07; (−0.53–0.68)	1.26; (−0.20–2.69)
	Sub-elite	9	7.88	9.38	0.15	0.06	0.34	0.07; (0.00–0.30)	0.44; (0.05–0.83)	−0.40; (−1.23–0.42)
	Physically active	7.61	7.53	6.84	0.66	0.67	0.38	0.004; (0.00–0.06)	0.06; (−0.23–0.35)	0.34; (−0.42–1.09)

LA—lactate; *MCT2*—monocarboxylate transporter 2; LArest—LA concentration before the warm-up at rest; LA30′—LA at 30′ min after the second Wingate test; LAmax—maximum LA concentration; ACC—LA accumulation capacity (the difference between LAmax and LArest); DCC—delta clearance capacity (the difference between LAmax and LA30); * false discovery rate (FDR) correction; CI—confidence interval; AA—homozygote for the major allele (A)**;** AT—heterozygote; TT—homozygote for the minor allele (T).

**Table 4:** Blood LA concentrations (mmol/L) in all, elite, sub-elite, and physically active individuals at rest, after exercise, and during recovery across genotypes in the *MCT2* rs995343 polymorphism, analyzed using codominant, dominant, and recessive allele models, along with their effects (η^2^, d) with 95% CI and post hoc power.

		Genotype			Models			Effect (η^2^, d); (95% CI); Post Hoc Power		
		AA	AG	GG	Codominant	Dominant	Recessive	Codominant	Dominant	Recessive
					AA vs. AG vs. GG	AA vs. AG + GG	AA + AG vs. GG	AA vs. AG vs. GG	AA vs. AG + GG	AA + AG vs. GG
LArest	All	2.10	2.40	2.30	0.03/0.04 *	0.01/0.03 *	0.94	0.02; (0.00–0.11)	−0.31; (−0.54–−0.08)	−0.01; (−0.29–0.27)
	Elite	1.98	2.18	2.12	0.72	0.43	0.98	0.02; (0.00–0.23)	−0.26; (−0.91–0.38)	−0.01; (−0.95–0.92)
	Sub-elite	1.93	2.29	2.46	0.1	0.04/0.12 *	0.25	0.05; (0.00–0.24)	−0.44; (−0.87–−0.02)	−0.33; (−0.90–0.23)
	Physically active	2.23	2.51	2.27	0.12	0.15	0.37	0.02; (0.00–0.14)	−0.23; (−0.55–0.08)	0.16; (−0.19–0.51)
LA30′	All	8.42	8.54	8.05	0.53	0.99	0.28	0.004; (0.00–0.04)	0.00; (−0.23–0.24)	0.15; (−0.13–0.44)
	Elite	9.56	9.44	10.57	0.68	0.92	0.38	0.02; (0.00–0.24)	−0.03; (−0.67–0.61)	−0.42; (−1.36–0.52)
	Sub-elite	9.05	9.6	8.25	0.32	0.68	0.2	0.02; (0.00–0.17)	−0.09; (−0.51–0.33)	0.37; (−0.20–0.94)
	Physically active	7.77	7.7	7.67	0.98	0.86	0.91	0; (0.00–0.00)	0.03; (−0.28–0.34)	0.02; (−0.33–0.37)
LAmax	All	16.17	16.49	16.68	0.47	0.26	0.43	0.004; (0.00–0.05)	−0.13; (−0.37–0.10)	−0.11; (−0.39–0.17)
	Elite	18.41	18.27	19.04	0.79	0.99	0.51	0.01; (0.00–0.20)	0.004; (−0.64–0.65)	−0.32; (−1.26–0.62)
	Sub-elite	17.6	17.88	17.86	0.89	0.64	0.92	0.002; (0.00–0.06)	−0.10; (−0.52–0.32)	−0.03; (−0.59–0.54)
	Physically active	14.77	15.25	15.98	0.03/0.04 *	0.05	0.02/0.04 *	0.04; (0.00–0.18)	−0.32; (−0.63–−0.00)	−0.41; (−0.76–−0.06)
ACC	All	14.07	14.09	14.38	0.75	0.78	0.45	0.002; (0.00–0.03)	−0.03; (−0.27–0.20)	−0.11; (−0.39–0.17)
	Elite	16.43	16.08	16.93	0.66	0.76	0.45	0.02; (0.00–0.25)	0.10; (−0.54–0.74)	−0.36; (−1.30–0.58)
	Sub-elite	15.68	15.59	15	0.95	0.83	0.77	0.001 (0.00–0.03)	0.05 (−0.37–0.46)	0.08; (−0.48–0.65)
	Physically active	12.54	12.74	13.71	0.02/0.03 *	0.17	0.01/0.03 *	0.04; (0.00–0.18)	−0.22; (−0.53–0.09)	−0.48; (−0.84–−0.13)
DCC	All	7.75	7.95	8.63	0.05	0.17	0.02/0.06 *	0.02; (0.00–0.10)	−0.16; (−0.40–0.07)	−0.33; (−0.62–−0.05)
	Elite	8.86	8.83	8.47	0.95	0.91	0.76	0.002; (0.00–0.06)	0.04; (−0.60–0.68)	0.15; (−0.79–1.08)
	Sub-elite	8.55	8.28	9.61	0.12	0.99	0.05	0.04 (0.00–0.23)	0.003; (−0.42–0.42)	−0.57; (−1.14–−0.002)
	Physically active	7	7.55	8.31	0.01/0.02 *	0.03/0.03 *	0.01/0.02 *	0.04; (0.003–0.19)	−0.36; (−0.67–−0.04)	−0.44; (−0.80–−0.09)

*LA*—lactate; MCT2—monocarboxylate transporter 2; LArest—LA concentration before the warm-up at rest; LA30′—LA at 30′ min after the second Wingate test; LAmax—maximum LA concentration; ACC—LA accumulation capacity (the difference between LAmax and LArest); DCC—delta clearance capacity (the difference between LAmax and LA30′); * false discovery rate (FDR) correction; CI—confidence interval; *AA*—homozygote for the major allele (A); *AG*—heterozygote; *GG*—homozygote for the minor allele (G).2.3. MCT2 rs3763979.

**Table 5:** Blood LA concentrations (mmol/L) in all, elite, sub-elite, and physically active individuals at rest, after exercise, and during recovery across genotypes in the *MCT2* rs3763979 polymorphism, analyzed using codominant, dominant, and recessive allele models, along with their effects (η^2^, d) with 95% CI and post hoc power.

		Genotype			Models			Effect (η^2^, d); (95% CI); Post Hoc Power		
		CC	CT	TT	Codominant	Dominant	Recessive	Codominant	Dominant	Recessive
					CC vs. CT vs. TT	CC vs. CT + TT	CC + CT vs. TT	CC vs. CT vs. TT	CC vs. CT + TT	CC + CT vs. TT
LArest	All	2.3	2.16	3.28	0.67	0.76	0.003/0.009 *	0.03; (0.01–0.13)	0.04; (−0.22–0.29)	−1.14; (−1.89–−0.38)
	Elite	2.09	2.11	-	-	-	-	-	-	-
	Sub-elite	2.23	2.07	2.25	0.75	0.47	0.94	0.006; (0.00–0.10)	0.17; (−0.29–0.62)	−0.06; (−1.46–1.34)
	Physically active	2.37	2.24	3.69	0.003/0.005 *	0.8	0.001/0.003 *	0.06; (0.01–0.22)	−0.05; (−0.39–0.30)	−1.51; (−2.41–−0.60)
LA30′	All	8.13	9.33	9.84	0.67	0.001/0.003 *	0.19	0.03; (0.01; 0.14)	−0.44; (−0.70–−0.18)	−0.51; (−1.26–0.24)
	Elite	10.22	9.4	-	-	-	-	-	-	-
	Sub-elite	8.92	10.15	12.13	0.11	0.06	0.19	0.04; (0.00–0.24)	−0.45; (−0.91–0.01)	−0.94; (−2.35–0.47)
	Physically active	7.46	8.59	8.92	0.03/0.04 *	0.01/0.03 *	0.29	0.04; (0.00–0.18)	−0.46; (−0.81–−0.12)	−0.48; (−1.37–0.41)
LAmax	All	16.3	16.87	16.68	0.67	0.12	0.81	0.007; (0.00–0.06)	−0.20; (−0.45–0.06)	−0.09; (−0.84–0.66)
	Elite	18.73	18.3	-	-	-	-	-	-	-
	Sub-elite	17.64	17.97	21.78	0.1	0.31	0.04/0.12 *	0.05; (0.00–0.24)	−0.24; (−0.70–0.22)	−1.52; (−2.93–−0.10)
	Physically active	15.19	15.65	14.64	0.43	0.37	0.53	0.009; (0.00–0.09)	−0.16; (−0.50–0.19)	0.29; (−0.60–1.18)
ACC	All	14	14.7	13.4	0.67	0.1	0.48	0.01; (0.00–0.08)	−0.21; (−0.47–0.04)	0.27; (−0.48–1.02)
	Elite	16.63	16.18	-	-	-	-	-	-	-
	Sub-elite	15.41	15.91	19.53	0.08	0.2	0.04/0.12 *	0.05; (0.00–0.25)	−0.30; (−0.76–0.16)	−1.52; (−2.93–−0.10)
	Physically active	12.82	13.41	10.96	0.05	0.43	0.05	0.03; (0.00–0.17)	−0.14; (−0.48–0.20)	0.90; (0.01–1.80)
DCC	All	8.17	7.53	6.84	0.67	0.02/0.06 *	0.17	0.02; (0.00–0.10)	0.31; (0.05–0.57)	0.53; (−0.23–1.28)
	Elite	8.5	8.9	-	-	-	-	-	-	-
	Sub-elite	8.72	7.83	9.65	0.19	0.15	0.48	0.03; (0.00–0.21)	0.34; (−0.12–0.80)	−0.51; (−1.91–0.89)
	Physically active	7.73	7.07	5.72	0.04/0.06 *	0.03/0.06 *	0.06	0.03; (0.00–0.17); 0.8	0.38; (0.04–0.73)	0.87; (−0.03–1.76)

LA—lactate; *MCT2*—monocarboxylate transporter 2; LArest—LA concentration before the warm-up at rest; LA30′—LA at 30′ min after the second Wingate test; LAmax—maximum LA concentration; ACC—LA accumulation capacity (the difference between LAmax and LArest); DCC—delta clearance capacity (the difference between LAmax and LA30′); * false discovery rate (FDR) correction; CI—confidence interval; CC—homozygote for the major allele (C), CT—heterozygote, TT—homozygote for the minor allele (T).

**Table 6:** Blood LA concentrations (mmol/L) in all, elite, sub-elite, and physically active individuals at rest, after exercise, and during recovery across genotypes in the *MCT4* rs11323780 polymorphism, analyzed using the codominant, dominant, and recessive allele models, along with their effects (η^2^, d) with 95% CI and post hoc power.

		Genotype			Models			Effect (η^2^, d); (95% CI); Post Hoc Power		
		--	T-	TT	Codominant	Dominant	Recessive	Codominant	Dominant	Recessive
					-- vs. T- vs. TT	-- vs. T- + TT	-- + T- vs. TT	-- vs. T- vs. TT	-- vs. T- + TT	-- + T- vs. TT
LArest	All	2.35	2.35	2.09	0.07	0.49	0.02/0.06 *	0.02; (0.00–0.09)	0.08; (−0.15–0.32)	0.30; (0.04–0.55)
	Elite	2.31	2.33	1.62	0.04/0.06 *	0.26	0.01/0.03 *	0.2; (0.00–0.51); 0.8	0.39; (−0.28–1.07)	0.96; (0.21–1.69); 0.8
	Sub-elite	2.38	2.22	1.98	0.24	0.27	0.14	0.03; (0.00–0.19)	0.27; (−0.18–0.72)	0.33; (−0.11–0.76)
	Physically active	2.28	2.34	2.44	0.61	0.67	0.47	0.005; (0.00–0.07)	−0.07; (−0.38–0.24)	0.13; (−0.23–0.49)
LA30′	All	8.47	8.12	9	0.09	0.49	0.05	0.01; (0.00–0.09)	0.03; (−0.21–0.27)	−0.26; (−0.52–−0.00)
	Elite	10.11	9.29	9.66	0.69	0.43	0.95	0.02; (0.00–0.24)	0.27; (−0.40–0.94)	−0.02; (−0.73–0.69)
	Sub-elite	9.43	9.03	9.44	0.8	0.73	0.69	0.004; (0.00–0.08)	0.08; (−0.37–0.52)	−0.09; (−0.52–0.35)
	Physically active	8.49	7.68	7.43	0.1	0.9	0.04/0.12 *	0.02; (0.00–0.14)	−0.02; (−0.33–0.29)	−0.38; (−0.74–−0.02)
LAmax	All	16.36	16.27	16.86	0.29	0.49	0.12	0.007; (0.00–0.06)	−0.04; (−0.28–0.20)	−0.20; (−0.46–0.05)
	Elite	18.94	18.6	17.39	0.24	0.34	0.1	0.07; (0.00–0.39)	0.33; (−0.35–1.00)	0.61; (−0.12–1.33)
	Sub-elite	17.61	17.86	17.83	0.93	0.7	0.94	0.001; (0.00–0.04)	−0.09; (−0.53–0.36)	−0.02; (−0.45–0.42)
	Physically active	16	15.22	15	0.06	0.85	0.02/0.06 *	0.03; (0.00–0.16)	−0.03; (−0.34–0.28)	−0.42; (−0.78–−0.06)
ACC	All	14.01	13.92	14.77	0.07	0.49	0.02/0.06 *	0.02; (0.00–0.09)	−0.06; (−0.30–0.17)	−0.30; (−0.56–−0.04)
	Elite	16.63	16.37	15.77	0.59	0.5	0.33	0.03; (0.00–0.27)	0.23; (−0.44–0.90)	0.36; (−0.36–1.07)
	Sub-elite	15.24	15.64	15.85	0.7	0.43	0.56	0.007 (0.00–0.11)	−0.18; (−0.63–0.27)	−0.13 (−0.56–0.31)
	Physically active	13.72	12.88	12.56	0.02/0.03 *	0.99	0.01/0.03 *	0.04; (0.00–0.19)	−0.00; (−0.31–0.31)	−0.48; (−0.84–−0.12)
DCC	All	7.88	8.15	7.86	0.54	0.49	0.52	0.004; (0.00–0.04)	−0.08; (−0.32–0.16)	0.08; (−0.17–0.34)
	Elite	8.83	9.31	7.73	0.25	0.96	0.11	0.07; (0.00–0.38)	0.02; (−0.65–0.69)	0.59; (−0.14–1.31)
	Sub-elite	8.18	8.83	8.38	0.43	0.33	0.64	0.02; (0.00–0.15)	−2.22; (−0.67–0.23)	0.10; (−0.33–0.54)
	Physically active	7.52	7.54	7.57	0.99	0.97	0.92	0; (0.00–0.00)	−0.01; (−0.32–0.30)	0.02; (−0.34–0.37)

LA—lactate; *MCT4*—monocarboxylate transporter 4; LArest—LA concentration before the warm-up at rest; LA30′—LA at 30′ min after the second Wingate test; LAmax—maximum LA concentration; ACC—LA accumulation capacity (the difference between LAmax and LArest); DCC—delta clearance capacity (the difference between LAmax and LA30′); * false discovery rate (FDR) correction; CI—confidence interval; --—homozygote for the major allele; T-—heterozygote; TT—homozygote for the minor allele (T).

**Table 7:** Pearson’s linear correlation between average LA concentration and ACC, LAmax, and DCC concentrations.

	LArest	LAaftwarm	LABETWEEN	LAaft2WAnt	LA3′	LA6′	LA9′	LA20′	LA30′	LAmax	ACC	DCC
LArest	1.00	0.28	0.16	0.10	0.16	0.15	0.06	0.06	0.08	0.16		0.08
LAaftwarm	0.28	1.00	0.38	0.33	0.33	0.32	0.26	0.26	0.26	0.33	0.24	0.07
LABETWEEN	0.16	0.38	1.00	0.78	0.80	0.75	0.72	0.61	0.50	0.77	0.72	0.30
LAaft2WAnt	0.10	0.33	0.78	1.00	0.75	0.71	0.67	0.53	0.43	0.72	0.69	0.32
LA3′	0.16	0.33	0.80	0.75	1.00	0.92	0.86	0.73	0.60	0.95	0.90	0.39
LA6′	0.15	0.32	0.75	0.71	0.92	1.00	0.93	0.81	0.69	0.97	0.92	0.30
LA9′	0.06	0.26	0.72	0.67	0.86	0.93	1.00	0.85	0.75	0.93	0.91	0.17
LA20′	0.06	0.26	0.61	0.53	0.73	0.81	0.85	1.00	0.93	0.79	0.77	−0.22
LA30′	0.08	0.26	0.50	0.43	0.60	0.69	0.75	0.93	1.00	0.67	0.64	
LAmax	0.16	0.33	0.77	0.72	0.95	0.97	0.93	0.79	0.67	1.00		
ACC		0.24	0.72	0.69	0.90	0.92	0.91	0.77	0.64		1.00	0.33
DCC	0.08	0.07	0.30	0.32	0.39	0.30	0.17	−0.22			0.33	1.00

LArest—LA concentration before the warm-up at rest; LAaftwarm—LA after warm-up; LABETWEEN—LA in the third minute of rest after the first but before the second Wingate test; LAaft2Want—LA immediately after the second Wingate test; LA3′—LA at 3′ min post-second Wingate; LA6′—LA at 6′ min post-second Wingate, LA9′—LA at 9′ min post-second Wingate, LA20′—LA at 20′ min post-second Wingate; LA30′—LA at 30′ min after the second Wingate test; LAmax—maximum LA concentration; ACC—LA accumulation capacity (the difference between LAmax and LArest); DCC—delta clearance capacity (the difference between LAmax and LA30′).

## Data Availability

The data for this study are available in the European Variation Archive (EVA) at EMBL-EBI, under accession number PRJEB85239 (https://www.ebi.ac.uk/eva/?eva-study=PRJEB85239) (accessed on 28 January 2025).

## Supplementary Materials

Supporting information can be downloaded at the following link: https://www.mdpi.com/article/10.3390/ijms26167865/s1.
